# Adipose-derived stem cell exosomes: from functional mechanisms to clinical translation in diabetic foot ulcer management

**DOI:** 10.3389/fendo.2026.1795114

**Published:** 2026-05-29

**Authors:** Xintong Zhong, Guohui Wu

**Affiliations:** 1 The Affiliated Eye Hospital, Jiangxi Medical College, Nanchang University, Nanchang, China; 2 School of Optometry and Ophthalmology, Jiangxi Medical College, Nanchang University, Nanchang, China; 3 Jiangxi Clinical Research Center for Ophthalmic Disease, Nanchang, China; 4 Jiangxi Provincial Key Laboratory of Vitreoretinal Diseases for Health, Nanchang, China; 5 Jiangxi Province Key Laboratory of Ophthalmology and Vision Sciences, Nanchang, China

**Keywords:** adipose-derived stem cell exosomes, cell-free therapeutic strategy, diabetic ulcer, tissue regeneration, wound healing

## Abstract

Diabetes is a major global public health challenge. Diabetic foot ulcer (DFU), one of its most severe complications, imposes a substantial economic burden and severely impairs patients’ quality of life. Current treatment strategies often yield suboptimal outcomes, underscoring the urgent need for novel therapeutic approaches. Adipose-derived stem cell exosomes (ADSC-Exos) have emerged as a promising cell-free therapy with significant clinical potential. This review integrates the mechanisms through which ADSC-Exos facilitate diabetic wound healing with the phases of wound healing. It elucidates that ADSC-Exos function by alleviating oxidative stress, modulating inflammation, promoting angiogenesis, stimulating cell proliferation and migration, and suppressing excessive fibrosis. Additionally, this study organizes insights into how delivery strategies based on smart materials (e.g., hydrogels and bioscaffolds) and the latest physical modalities can overcome bottlenecks in clinical translation. This review aims to provide an integrated perspective for developing next-generation DFU therapies.

## 1 Introduction

Diabetes mellitus constitutes a profound global health burden, with its prevalence projected to reach 10.9% by 2045 (1). Consequently, up to 34% of diabetic patients will develop diabetic foot ulcers (DFUs) over their lifetime. The clinical trajectory of DFUs is remarkably severe; nearly one-fifth of affected individuals ultimately require lower-extremity amputation (2), accompanied by a striking five-year mortality rate approaching 50% following initial diagnosis (3, 4).The pathogenesis of DFUs is inherently multifactorial. Persistent hyperglycemia acts as the primary driver. Once initiated, the condition is severely compounded by a cascade of secondary issues: peripheral neuropathy, microvascular dysfunction, profound oxidative stress, and chronic immune dysregulation (5–9).

Current clinical standards of care include surgical debridement, specialized wound dressings, pressure off-loading, and vascular reconstruction (10) (To better highlight the translational potential of ADSC-Exos, a detailed comparison with current mainstream DFU therapies—regarding mechanisms of action, core advantages, limitations, and clinical status—is presented in **Table 1**.); However, the therapeutic outcomes of these conventional treatments remain unsatisfactory. This limitation highlights the urgent need for new and highly effective therapeutic approaches (11). In this context, stem cell therapies have demonstrated robust regenerative efficacy. Transplanted stem cells orchestrate wound repair by secreting paracrine factors. These factors subsequently drive cell migration, immunomodulation, extracellular matrix (ECM) remodeling, and angiogenesis (12, 13). Nevertheless, direct cell transplantation is critically hindered by translational bottlenecks, including poor *in vivo* engraftment, inherent immunogenicity, ethical constraints, and potential tumorigenicity (14).

**Table 1:** Comparison of ADSC-Exos with current therapies for DFU.

Therapy	Mechanism of action	Core advantages	Limitations	Current clinical status	References
ADSC-Exos	Alleviating oxidative stress, modulating inflammation, promoting angiogenesis, Stimulating cell proliferation and migration, and suppressing excessive fibrosis.	Low immunogenicity, no tumorigenic risk; Stable and easy to store (can be lyophilized); Lipid bilayer protects contents from protease degradation.	Lack of universally standardized extraction and purification protocols; High cost for large-scale GMP production; Exact molecular regulatory networks require further elucidation.	Mostly in animal studies with high translational potential	(25–28)
Stem Cells	Paracrine secretion of growth factors and cytokines; limited multilineage differentiation potential; immunomodulation.	Continuously responds to the microenvironment to secrete various active factors; Fundamentally improves the ulcer microenvironment	Live-cell defects: Extremely low survival rate in the hostile DFU microenvironment (high glucose, hypoxia, high ROS); Potential tumorigenic and immune rejection risks; Strict cold chain storage requirements.	Still transitioning from clinical exploration to routine application due to insufficient standardized protocols and high-level evidence.	(29–32)
PRP	Releases a high concentration of autologous growth factors (e.g., PDGF, TGF-β, VEGF) upon activation; forms a fibrin scaffold to promote cell migration.	Autologous origin (no immune rejection or ethical controversies); Simple preparation, relatively low cost; Point-of-care (POC) applicability.	Restricted by patient status: Impaired platelet function in diabetic patients leads to poor-quality factors; Short half-life of released factors; High inter-individual variability in efficacy.	Widely used: Commonly used as an adjuvant therapy, but efficacy depends heavily on the patient’s baseline health.	(33, 34)
Growth Factors (e.g., rhPDGF)	Binds to specific cell surface receptors, activates intracellular signaling pathways, and directly stimulates angiogenesis or cell proliferation.	Clear therapeutic targets; Standardized dosing; FDA-approved commercial products available (e.g., Becaplermin).	Very short half-life: Easily degraded by high concentrations of proteases (e.g., MMPs) in DFU exudates; Requires frequent, large-dose applications; Black-box warning for increased cancer risk at high doses.	Standard treatment option: Application is limited by high cost, short duration of efficacy, and potential safety concerns.	(35, 36)
NPWT	Physical mechanism: Removes exudate and necrotic tissue via suction; reduces edema; mechanically stimulates granulation tissue growth; decreases local bacterial load.	Definitive physical debridement effect; Avoids systemic side effects associated with biologics; Effective management of highly exudative wounds.	Cannot correct diabetes-induced cellular senescence and dysfunction at the molecular level; Requires wearing a device, restricting patient mobility; Contraindicated for severely ischemic wounds or exposed bone/tendon.	Routine therapy: First-line physical adjuvant therapy for complex DFUs, usually combined with biologics.	(37, 38)

ADSC-Exos, Adipose-derived stem cell exosomes; ADSCs, Adipose-derived stem cells; BM-MSCs, Bone marrow-derived mesenchymal stem cells; DFU, Diabetic foot ulcer; FDA, Food and Drug Administration; GMP, Good manufacturing practice; MMPs, Matrix metalloproteinases; NPWT, Negative pressure wound therapy; PDGF, Platelet-derived growth factor; POC, Point-of-care; PRP, Platelet-rich plasma; rhPDGF, Recombinant human platelet-derived growth factor; ROS, Reactive oxygen species; TGF-β, Transforming growth factor-beta; VEGF, Vascular endothelial growth factor.

**Figure 1:**
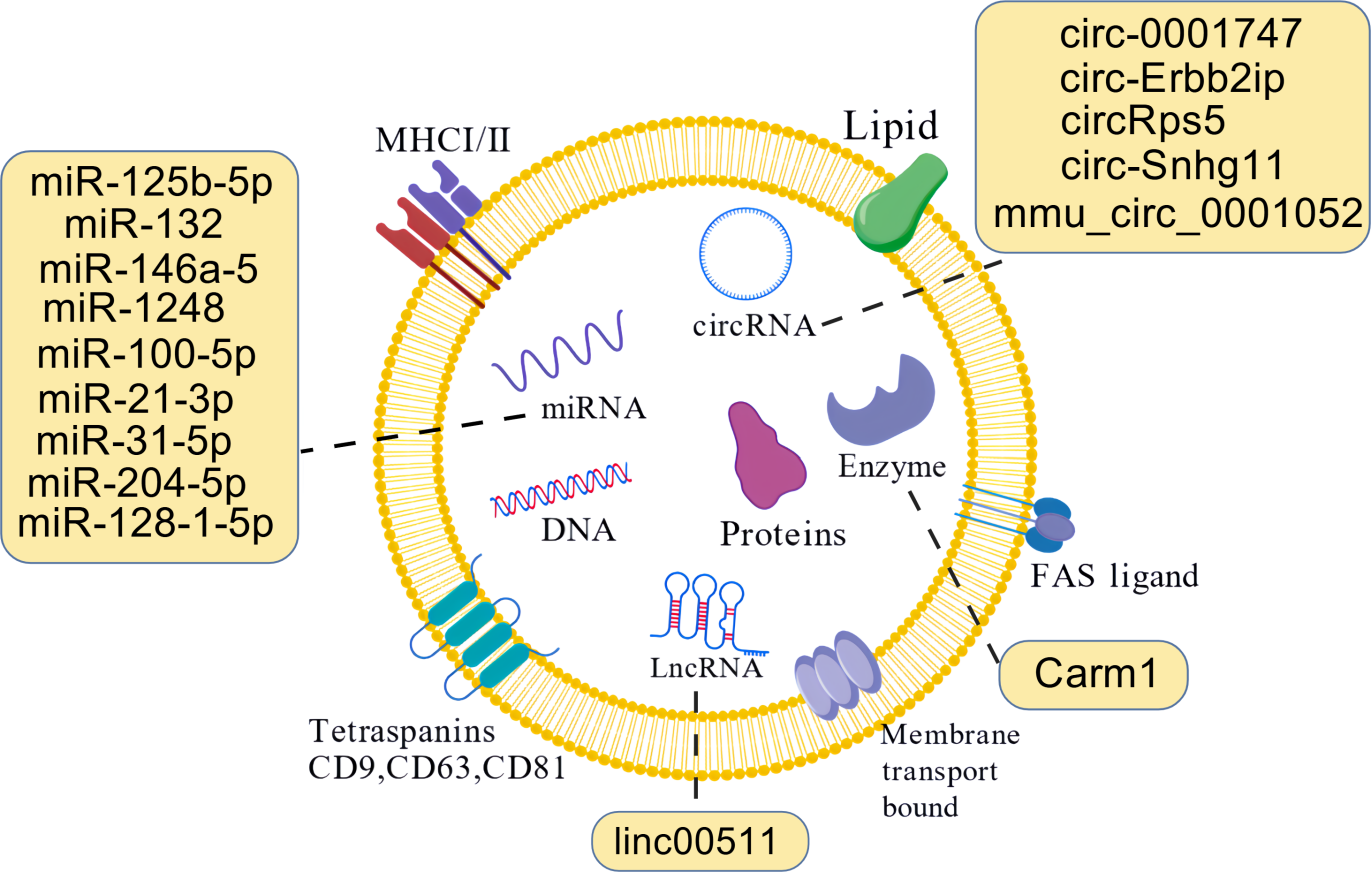
The structure and cargoes of ADSC-Exos. Exosomes derived from adipose-derived stem cells share a typical nano-scale bilayer vesicle structure with those from other cell types. Their core architecture consists of a phospholipid bilayer, surface proteins (including transmembrane proteins such as CD9/CD63/CD81, adhesion proteins, immunomodulatory molecules like MHC I/II, FAS ligand, and membrane transport proteins), as well as lipids. These exosomes carry functional cargo both on their surface and within their lumen, primarily comprising nucleic acids (e.g., miRNA, DNA, LncRNA, circRNA), proteins, and lipids. Labels in the figure highlight key cargo molecules emphasized in this study, such as miR-125b-5p and the enzyme Carm1. (Schematic created with BioGDP). Carm1, Coactivator-associated arginine methyltransferase 1.

These systemic limitations have driven the advancement of cell-free therapeutics, particularly those targeting adipose-derived stem cell extracellular vesicles (ADSC-EVs) (15, 16). EVs are naturally occurring lipid vesicles that cells use to communicate. Because they carry bioactive molecules from their parent cells, they retain similar regenerative and immunomodulatory properties. Their lipid bilayer also makes them excellent natural nano-delivery vehicles. This is especially important for diabetic foot ulcers, where the lipid membrane protects fragile RNAs and proteins from rapid breakdown in the harsh wound environment. Within the EV family, exosomes are a specific subgroup measuring 40 to 150 nm. Originating from the endosomal pathway, their main role is to act as delivery shuttles. They enter recipient cells, such as macrophages and fibroblasts, to release their contents and directly activate healing mechanisms (17, 18). Stem cell exosomes are pivotal mediators of the biological effects of paracrine factors from stem cells, providing an ideal approach for further cell-free therapeutic wound healing (19). They coordinate tissue regeneration by encapsulating and transferring a complex cargo of bioactive molecules—comprising regulatory proteins, lipids, and diverse functional nucleic acids, as depicted in **Figure 1** (20–22). Consequently, mesenchymal stem cell-derived exosomes effectively recapitulate the regenerative capabilities of their parent cells while safely circumventing the viability and oncogenic concerns associated with live-cell administration (23).

Although therapeutic exosomes originate from diverse cellular lineages, adipose-derived stem cell exosomes (ADSC-Exos) possess definitive advantages for DFU intervention. The ubiquitous abundance and minimally invasive procurement of adipose tissue significantly facilitate the scalable clinical translation of ADSC-Exos platforms (24). Importantly, while current literature reports promising outcomes for ADSC-Exos therapies, early attention must be paid to their fundamental limitations. A key inherent limitation lies in the substantial heterogeneity and cargo variability of ADSC-Exos, which are strongly influenced by donor traits, cell culture microenvironments, and isolation techniques. Such variability complicates the standardization of dosing and consistency of therapeutic effects, creating a notable barrier to clinical translation. We also critically highlight another major gap in the field: the overreliance on preclinical *in vitro* and rodent models, paired with a marked lack of robust human clinical trials (25). Unlike general mechanistic summaries, this review details the precise roles of specific ADSC-Exo molecules throughout the different phases of wound healing. We place a special focus on how these vesicles prevent excessive fibrosis to promote proper tissue repair. To address the current bottlenecks in clinical translation, we also evaluate a combined engineering approach. This strategy pairs exosome optimization via cellular preconditioning with advanced delivery methods. Ultimately, this review aims to provide a clear and practical roadmap for developing effective, cell-free therapies for diabetic foot ulcers.

## 2 Multiple therapeutic mechanisms of ADSC-Exos

Wound repair is classically simplified into four main phases: hemostasis, inflammation, proliferation, and dermal remodeling (39). However, in the pathological environment of DFU, this orderly progression is disrupted, leading to chronicity (40). Although contemporary literature frequently segregates the therapeutic actions of ADSC-Exos into discrete antioxidant, anti-inflammatory, and pro-angiogenic functions, these processes operate as a deeply intertwined continuum within the diabetic wound microenvironment. A critical synthesis of existing data indicates that ADSC-Exos orchestrate a highly hierarchical pathological cascade, wherein resolving upstream dysfunctions intrinsically propagates downstream regenerative phases.

**Figure 2:**
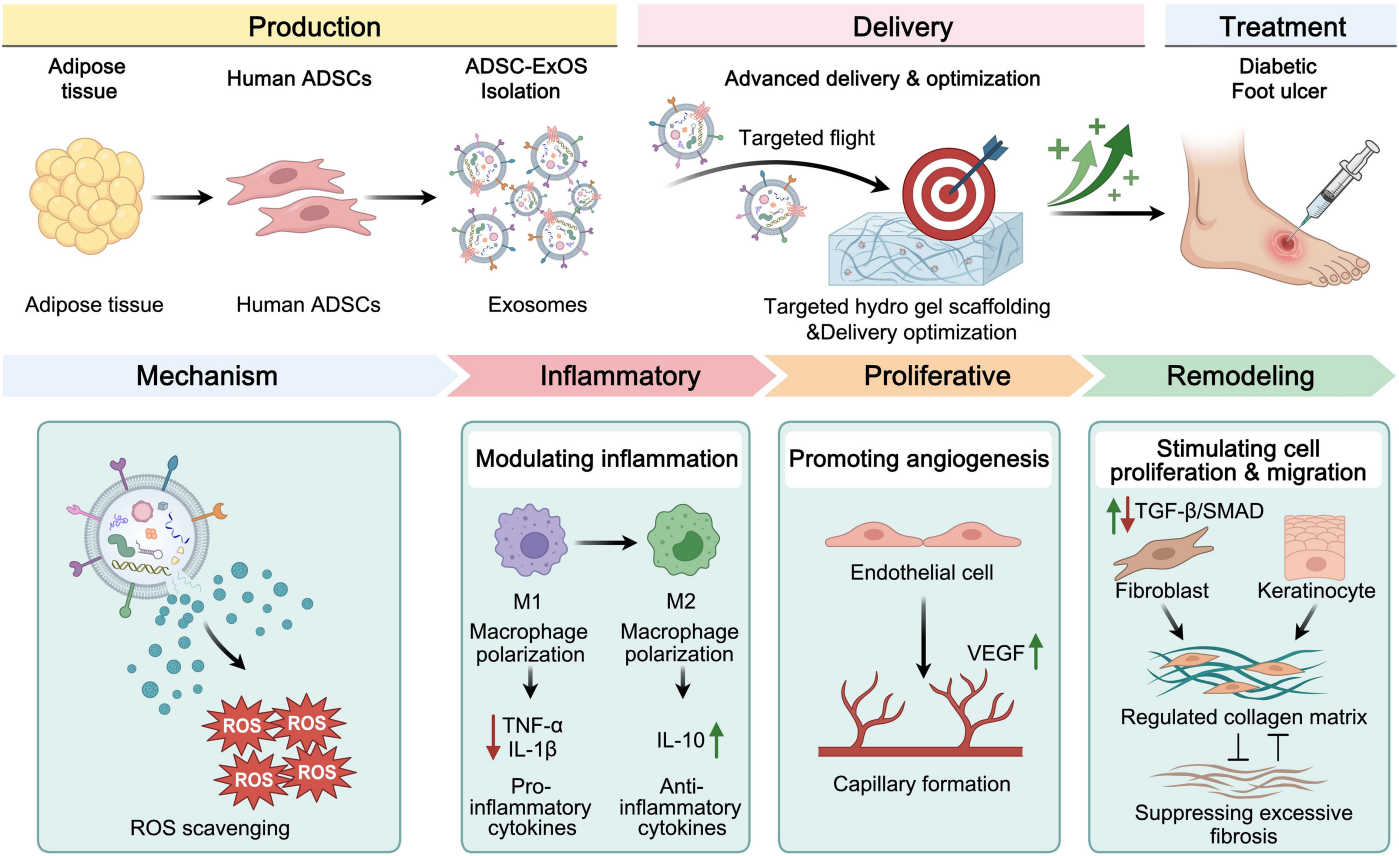
Mechanism of diabetic ulcer healing mediated by ADSC-Exos. The upper panel depicts the clinical translation workflow: human adipose-derived stem cells (ADSCs) are extracted to isolate ADSC-derived exosomes (ADSC-Exos), followed by advanced delivery and optimization (e.g., via targeted hydrogel scaffolding)for the treatment of diabetic foot ulcers. The lower panel illustrates how ADSC-Exos accelerate wound healing through coordinated multi-cellular actions and the delivery of diverse functional cargoes. These mechanisms involve reactive oxygen species (ROS) scavenging to alleviate oxidative stress and continuous interventions across three distinct healing phases (1): Inflammatory phase: ADSC-Exos modulate the inflammatory microenvironment by driving macrophage polarization from the pro-inflammatory M1 to the anti-inflammatory M2 phenotype, decreasing TNF-α and IL-1β while increasing IL-10 levels. (2)Proliferative phase: ADSC-Exos promote angiogenesis in endothelial cells by upregulating VEGF, thereby enhancing capillary formation. (3) Remodeling phase: ADSC-Exos stimulate the proliferation and migration of fibroblasts and keratinocytes via the regulation of the TGF-β/SMAD signaling pathway. This orchestrated regulation facilitates the formation of a regulated collagen matrix while suppressing excessive fibrosis to achieve high-quality healing. (Schematic created with Adobe Illustrator). ADSCs, adipose-derived stem cells; Exos, exosomes; ROS, reactive oxygen species; TNF-α, tumor necrosis factor-alpha; IL, interleukin; VEGF, vascular endothelial growth factor; TGF-β, transforming growth factor-beta.

To conceptualize this spatiotemporal synergy, we propose an integrated mechanistic framework, as delineated in **Figure 2**. Initially, persistent hyperglycemia drives profound oxidative stress, acting as the primary catalyst for chronic inflammation (41, 42). ADSC-Exos counteract this by reactivating endogenous antioxidant axes, notably the Keap1/Nrf2 signaling network (26). This targeted clearance of reactive oxygen species serves as an absolute biological prerequisite for silencing pro-inflammatory effectors like NF-κB, thereby driving macrophage repolarization from a cytotoxic M1 to an immunoregulatory M2 state (27, 43). This resolution of inflammation is mechanistically coupled to vascular recovery. Because prolonged M1 polarization in diabetic ulcers intrinsically paralyzes endothelial function (44), enforcing an M2-dominant microenvironment allows ADSC-Exos to alleviate this cellular suppression. This precise immunological shift activates VEGF and HIF-1α signaling cascades, ultimately rescuing robust angiogenesis (45–47). Finally, this proliferative momentum is strictly balanced by controlled tissue remodeling. While ADSC-Exos stimulate fibroblastic expansion to facilitate wound closure, they simultaneously impose tight epigenetic regulation over the TGF-β/Smad axis. This dual modulation guarantees that essential extracellular matrix deposition matures into an ordered architecture rather than progressing toward pathological fibrosis (28, 48).

Building on this framework, the following sections detail how specific ADSC-Exo cargoes actively intervene in the diabetic niche. We explore how these molecular signals shift the wound environment from a state of chronic degradation to one of active, functional repair. The key therapeutic mechanisms, active cargos, and underlying signaling pathways of ADSC-Exos discussed in this section are summarized in **Table 2**.

**Table 2:** The functions of ADSCs-derived EVs in diabetic foot ulcers.

Mechanism category	Active cargo	Target/pathway	Functions	Experimental evidence	Translational stage	Ref.
Alleviation of Oxidative Stress	circ-0001747	miR-199a-5p/HIF-1α	Alleviates high glucose-induced suppression of HIF-1α activity and reduces oxidative stress.	*In vitro* (Cell) & *In vivo* (Rodent)	Preclinical	(45)
	circ-Erbb2ip	miR-670-5p/Nrf1	Activates the Nrf1 pathway to ameliorate oxidative stress at the diabetic wound site.	*In vitro* (Cell) & *In vivo* (Rodent)	Preclinical	(51)
	miR-125b-5p	Keap1/Nrf2	Activates the Keap1/Nrf2 signaling axis, alleviating oxidative stress damage in fibroblasts.	*In vitro* (Cell) & *In vivo* (Rodent)	Preclinical	(26)
	Unspecified	SIRT3/SOD2	Upregulates SIRT3 to enhance antioxidant capacity and reduce mitochondrial damage.	*In vitro* (Cell) & *In vivo* (Rodent)	Preclinical	(52)
Inflammation Regulation	Carm1	IL-6, TNF-α, IL-10	Promotes nuclear translocation to inhibit pro-inflammatory and promote anti-inflammatory cytokines.	*In vitro* (Cell) & *In vivo* (Rodent)	Preclinical	(43)
	miR-132	NF-κB pathway	Promotes M2 macrophage polarization by inhibiting the NF-κB pathway.	*In vitro* (Cell) & *In vivo* (Rodent)	Preclinical	(27)
	circRps5	miR-124-3p	Drives macrophage polarization towards the M2 phenotype and alleviates excessive autophagy.	*In vitro* (Cell) & *In vivo* (Rodent)	Preclinical	(58)
	circ-Snhg11	miR-144-3p/HIF-1α	Promotes VEGF/STAT3 pathway activation and induces M2-like macrophage polarization.	*In vitro* (Cell) & *In vivo* (Rodent)	Preclinical	(46)
Promotion of Angiogenesis	miR-146a-5p	JAZF1/VEGFA	Activates vascular endothelial cell function and promotes VEGFA expression.	*In vitro* (Cell) & *In vivo* (Rodent)	Preclinical	(47)
	miR-1248	VEGF-A, Angpt-1	Improves angiogenesis and maturation by upregulating key pro-angiogenic factors.	*In vitro* (Cell) & *In vivo* (Rodent)	Preclinical	(63)
	mmu_circ_0001052	miR-106a-5p/FGF4	Activates the FGF4/p38MAPK pathway to promote angiogenesis.	*In vitro* (Cell) & *In vivo* (Rodent)	Preclinical	(64)
	linc00511	PAQR3/Twist1	Inhibits PAQR3-BTRC-Twist1 axis, promoting EPC function and angiogenesis.	*In vitro* (Cell) & *In vivo* (Rodent)	Preclinical	(65)
	miR-100-5p	VEGF/CD31	Enhances angiogenesis and suppresses inflammation in diabetic wounds.	*In vitro* (Cell) & *In vivo* (Rodent)	Preclinical	(66)
Stimulation of Cell Proliferation & Migration	miR-21-3p, miR-126-5p, miR-31-5p	PI3K/AKT	Promotes fibroblast proliferation, migration, and collagen synthesis directly.	*In vitro* (Cell) & *In vivo* (Rodent)	Preclinical	(71)
	Unspecified	NAMPT	Activates epidermal cell autophagy flow to restore proliferation and migration.	*In vitro* (Cell) & *In vivo* (Rodent)	Preclinical	(68)
	Unspecified	TGF-β/Smad3	Inhibits TGF-β1 expression and Smad2/3 phosphorylation, promoting “scar-free” healing.	*In vitro* (Cell) & *In vivo* (Rodent)	Preclinical	(69)
	Unspecified	Bax/Caspase-3	Inhibits mitochondrial apoptosis pathway to restore fibroblast proliferation and migration.	*In vitro* (Cell) & *In vivo* (Rodent)	Preclinical	(70)
Inhibition of Excessive Fibrosis	miR-204-5p	TGF-β1/Smad2/3	Inhibits TGF-β1 expression and Smad2/3 phosphorylation, promoting “scar-free” healing.	*In vitro* (Cell) & *In vivo* (Rodent)	Preclinical	(48)
	miR-128-1-5p	TGF-β1/Smad2/3	Inhibits the TGF-β1/Smad pathway, promoting wound healing while inhibiting excessive fibrosis.	*In vitro* (Cell) & *In vivo* (Rodent)	Preclinical	(28)

Keap1, Kelch-like ECH-associated protein 1; Nrf, Nuclear respiratory factor; SIRT3, Sirtuin 3; SOD2, Superoxide dismutase 2; Carm1, Coactivator-associated arginine methyltransferase 1; STAT3, Signal transducer and activator of transcription 3; JAZF1, Juxtaposed with another zinc finger protein 1; Angpt-1, Angiopoietin-1; PAQR3, Progestin and adipoQ receptor family member 3; BTRC, Beta-transducin repeat containing E3 ubiquitin protein ligase; EPC, Endothelial progenitor cell; NAMPT, Nicotinamide phosphoribosyltransferase.

### 2.1 Alleviation of oxidative stress

When the production of reactive oxygen species (ROS) is overloaded, surpassing the capacity of the reductive rheostat, mammalian cells undergo a series of oxidative damage termed oxidative stress (49). During normal wound healing, low levels of ROS play roles in regulating the inflammatory response, clearing pathogens, and promoting cellular signal transduction throughout the entire wound healing process (41). However, in DFUs, factors such as persistent hyperglycemia lead to excessive accumulation of ROS, inducing oxidative stress, which triggers inflammation through multiple pathways and exacerbates vascular and neuropathic complications. It is a core upstream mechanism driving various pathophysiological abnormalities in DFU (42, 50). Therefore, effectively inhibiting oxidative stress is considered a key strategy for promoting DFU healing. Wang et al. (45) demonstrated that a high-glucose environment suppresses HIF-1α activity, and that hypoxic preconditioned ADSC-Exos (HypADSC-Exos) can alleviate this suppression via circ-0001747. Similarly, Tang et al. (51) found that HypADSC-Exos deliver high levels of circ-Erbb2ip, which upregulates the expression of Nrf1 by adsorbing miR-670-5p, thereby ameliorating oxidative stress at the diabetic wound site and ultimately accelerating wound healing. Extending these findings beyond circRNAs, Tian et al. (26) showed that ADSC-Exos effectively activate the antioxidant defense system in fibroblasts. They achieve this primarily via the delivery of miR-125b-5p to modulate the Keap1/Nrf2 signaling axis. By directly targeting and suppressing Keap1 expression, this microRNA alleviates high-glucose-induced oxidative stress damage. This allows fibroblasts to restore their proliferation, migration, and collagen synthesis. The clinical result was clear: treated subjects showed a significantly reduced wound size at Day 14 compared to the untreated HG-control group. Further evidence was provided by Zhang et al. (52), who confirmed that ADSC-Exos upregulate SIRT3. This upregulation promotes the deacetylation of SOD2, enhances antioxidant capacity, and reduces both mitochondrial damage and inflammatory responses. *In vivo* experiments showed that by the 7th day, malondialdehyde (MDA) levels in the exosome group had decreased, while superoxide dismutase (SOD) and total antioxidant capacity (T-AOC) had increased. Through these combined mechanisms, ADSC-Exos ameliorate high glucose (HG)-induced vascular endothelial dysfunction, promote angiogenesis, and accelerate diabetic wound healing.

### 2.2 Inflammation regulation

Alleviating oxidative stress is prerequisite to resolving inflammation, reduced ROS levels directly foster a microenvironment conducive to M1-to-M2 macrophage polarization (53). The balance between M1 and M2 macrophage polarization critically governs the progression of inflammation (54). When infection or inflammation is severe enough to affect an organ, macrophages first exhibit an M1 phenotype, releasing TNF-α, IL-1β, IL-12, and IL-23 to resist the stimulus. However, the persistent activation of the M1 phenotype can lead to tissue damage. At this time, M2 macrophages secrete large amounts of IL-10 and TGF-β to suppress inflammation, contributing to tissue repair, remodeling, angiogenesis, and the maintenance of homeostasis (55–57). In normal wound healing, the macrophage phenotype typically shifts from M1 to M2 around day 3 after injury, whereas diabetic wounds are characterized by dysregulated polarization and a persistent dominance of M1 macrophages, resulting in excessive inflammation at the wound site that severely impairs healing (44). Therefore, regulating macrophage polarization should be an important target for controlling DFU. The experiment by Zhang et al. (43) demonstrated that upon uptake by macrophages, ADSC-Exos release Carm1 into the nucleus. This nuclear translocation of Carm1 inhibits IL-6/TNF-α transcription while promoting IL-10 transcription. This drives M2 macrophage polarization and resolves inflammation. In addition, Ge et al. (27) successfully prepared miR-132-exo derived from ADSCs overexpressing miR-132. Experiments showed that miR-132-exo can promote macrophage polarization toward the M2 phenotype by inhibiting the NF-κB pathway, thereby effectively alleviating the excessive inflammatory response, evidenced by a marked reduction in pro-inflammatory markers and a significant shift toward the M2 macrophage phenotype compared to controls. Notably, Yin et al. (58) demonstrated that ADSC-Exos, by carrying circRps5, act as a molecular sponge to specifically adsorb miR-124-3p, relieving the inhibition of downstream pro-repair target genes by miR-124-3p. This shift drives macrophages toward M2 polarization and alleviates excessive autophagy, ultimately accelerating diabetic wound healing. Furthermore, Shi et al. (46) showed that hypoxic preconditioned ADSC-Exos deliver circ-Snhg11 to adsorb miR-144-3p. This interaction relieves the inhibition on HIF-1α and subsequently promotes the activation of the VEGF/STAT3 signaling pathway. As a result, Endothelial Progenitor Cell (EPC) function is improved, and M2 macrophage polarization is induced. These combined cellular responses alleviate excessive inflammation under HG conditions and accelerate diabetic wound healing.

### 2.3 Promotion of angiogenesis

Following the resolution of the inflammatory phase, angiogenesis becomes the next critical bottleneck. In normal wound healing, angiogenesis is a crucial step, which typically occurs during the proliferative phase following the inflammatory phase (59). In DFU, HG drives chronic oxidative stress and unresolved inflammation. The resulting accumulation of ROS and pro-inflammatory cytokines impairs endothelial function and downregulates pro-angiogenic factors like VEGF, ultimately arresting angiogenesis. This leads to insufficient local supply of nutrients and oxygen, accumulation of metabolic waste, and impedes the subsequent formation and filling of nascent granulation tissue (60–62). Therefore, promoting angiogenesis is a highly promising approach to accelerate DFU healing and reduce the risk of amputation. To restore this impaired network, many ADSC-Exos therapies target the classical VEGF signaling. A study by Che et al. (47) demonstrated that ADSC-Exos, by carrying highly expressed miR-146a-5p, target and inhibit the JAZF1 gene, thereby activating vascular endothelial cell function, promoting VEGFA expression and collagen synthesis, and ultimately accelerating angiogenesis and healing in diabetic wounds. To investigate the mechanism, they performed miRNA sequencing on human umbilical vein endothelial cells (HUVECs) treated with ADSC-Exos and found that miR-146a-5p expression was significantly upregulated. Then, by transfecting miR-146a-5p mimics and inhibitors, they found that this miRNA is a key molecule mediating the pro-angiogenic effects of ADSC-Exos. Similarly, Jian et al. (63) constructed a diabetic mouse model and injected mice with engineered exosomes overexpressing miR-1248 to create the experimental group. Experiments proved that Exo-miR-1248 treatment accelerated wound closure in diabetic mice, promoted re-epithelialization, RT-qPCR and WB show that the mRNA and protein levels of VEGF-A, Angpt-1, and TGF-β are upregulated. Exo-miR-1248 group exhibited a significantly higher capacity to locally upregulate the expression of pro-angiogenic genes compared to the other groups. Beyond relying on classical VEGF pathways, exosomes can also stimulate angiogenesis through alternative bypass signaling. For instance, Liang et al. (64) found that delivering mmu_circ_0001052 via ADSC-derived exosomes adsorbs miR-106a-5p. This effectively relieves the inhibition on FGF4 and activates the FGF4/p38MAPK pathway. As a result, both angiogenesis and overall healing in diabetic wounds are accelerated. Furthermore, Qiu et al. (65) confirmed that HG leads to upregulation of PAQR3, which subsequently causes a decrease in Twist1 protein levels, resulting in impaired angiogenesis. linc00511-overexpressing ADSC exosomes can inhibit the PAQR3–BTRC–Twist1 axis, reduces Twist1 ubiquitination degradation. This promotes EPC function, enhances angiogenesis, and accelerates diabetic foot ulcer healing. Importantly, demonstrating the direct crosstalk between inflammatory resolution and vascular regeneration, Liu et al. (66) identified miR‐100‐5p as a key modulator of angiogenesis and inflammation. *In vitro*, HypADSC‐Exos enhanced human umbilical vein endothelial cell and fibroblast proliferation, migration, and tube formation. In a rat DFU model, HypADSC-Exos administration reduced ulcer size, increased angiogenesis (VEGF/CD31 expression), and decreased inflammatory markers (TNF‐α, IL‐6). Findings show that HypADSC-Exos enhance angiogenesis, suppress inflammation, and accelerate wound healing *in vitro* and *in vivo*.

### 2.4 Stimulation of cell proliferation and migration

During the wound healing process, cell migration and proliferation are tightly coupled and mutually reinforcing. Cells first migrate to the correct location, then expand their population through proliferation; this synergistic effect ultimately leads to the formation of nascent granulation tissue rich in capillaries and fibroblasts, which provides a healthy base for epithelialization and smoothly transitions to the tissue remodeling phase (67). The experiment by Ren et al. (68) showed that ADSC-Exos upregulate the expression of key genes such as NAMPT, activate the epidermal cell autophagy flow inhibited by the HG environment, thereby restoring their inhibited proliferation and migration functions, ADSC-Exos significantly increased the wound closure rate of HaCaT cells in a scratch assay under HG conditions. Additionally, Hsu et al. (69) found that locally applied ADSC-Exos can be taken up by macrophages and fibroblasts at the wound site. ADSC-Exos stimulates macrophages to secrete more TGF-β1, and the increased TGF-β1 activates the TGF-β/Smad3 signaling pathway in fibroblasts. Smad3 enters the nucleus and initiates the synthesis of ECM components such as Col-I and α-SMA, promoting fibroblast proliferation and differentiation into myofibroblasts. On the other hand, Yang et al. (70) proved that ADSC-Exos can effectively reduce oxidative stress damage and apoptosis in fibroblasts exposed to the HG microenvironment. They achieve this by inhibiting the Bax/Caspase-3-mediated mitochondrial apoptosis pathway. This inhibition successfully restores the fibroblasts’ capacity for proliferation, migration, and collagen synthesis. Ultimately, this cascade accelerates diabetic wound healing and improves overall healing quality in animal models. Wang et al. (71) found that HypADSC-Exos, by upregulating miR-21-3p, miR-126-5p, and miR-31-5p to activate the PI3K/AKT signaling pathway, directly promotes fibroblast proliferation, migration, and collagen synthesis, and accelerates high-quality healing of diabetic wounds.

### 2.5 Inhibition of excessive fibrosis

**Figure 3:**
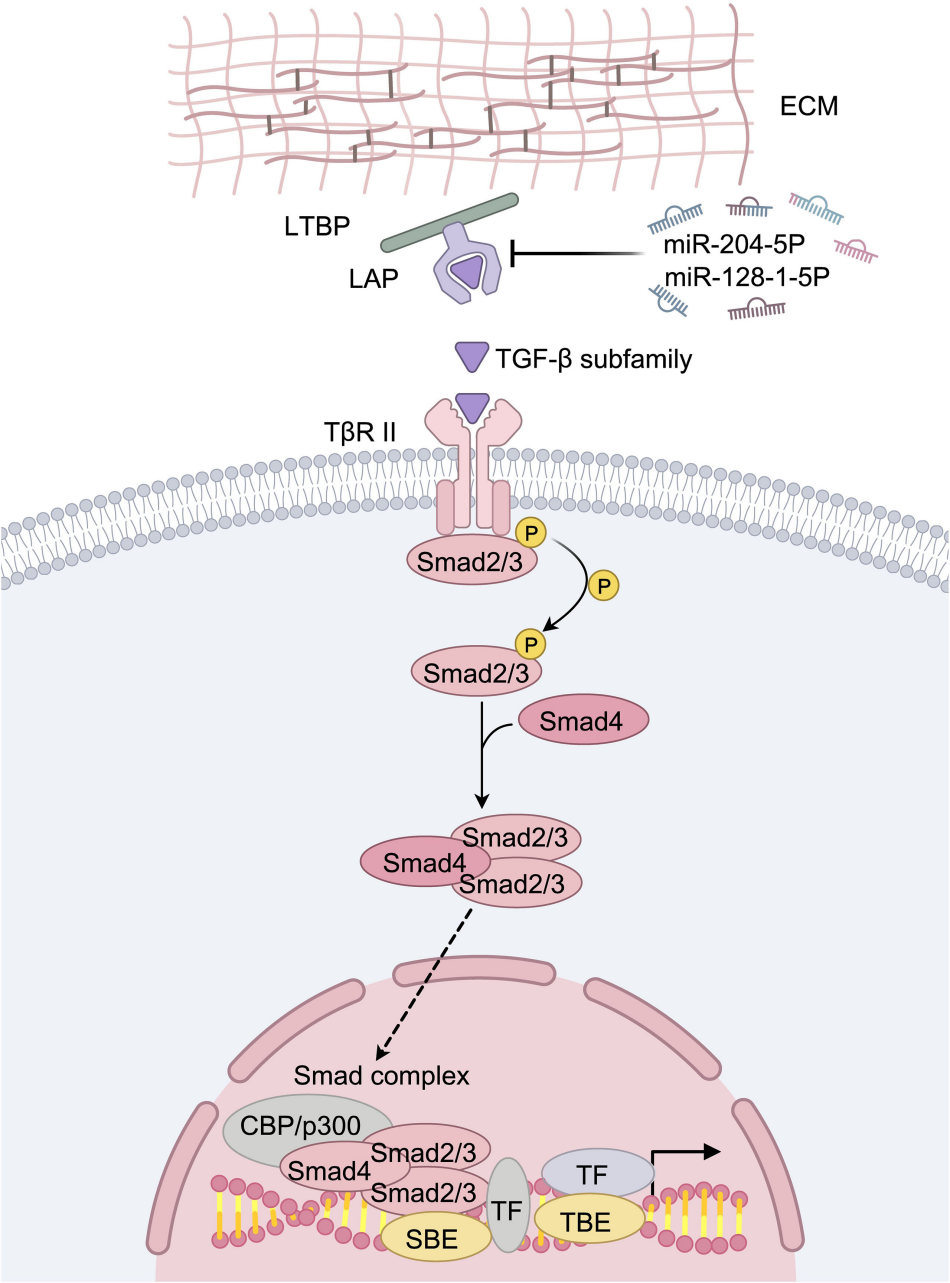
TGF-β/Smad signaling pathway. In the extracellular space, TGF-β is sequestered within the ECM as a latent complex composed of itself, the latency-associated peptide (LAP), and the latent TGF-β binding protein (LTBP). Upon activation, the liberated TGF-β ligand binds to the TGF-β type II receptor (TβRII) on the cell membrane. This engagement initiates intracellular signaling by phosphorylating the receptor-regulated Smads, Smad2 and Smad3. The phosphorylated Smad2/3 then forms a heteromeric complex with the common-mediator Smad4 (gray oval), which translocates into the nucleus. Within the nucleus, this Smad complex collaborates with specific transcription factors (TFs) and the transcriptional co-activators CBP/p300 to bind specific DNA response elements, such as the Smad binding element (SBE). This regulates the transcription of target genes. Additionally,miR-204-5P and miR-128-1-5P are indicated to potentially exert a negative regulatory effect on this pathway by targeting TGF-β1 mRNA for degradation. (Schematic created with Adobe Illustrator). LAP, Latency-associated peptide; LTBP, Latent TGF-β binding protein; TβRII, TGF-β type II receptor; CBP, CREB-binding protein; SBE, Smad binding element.

The promise of ADSC-Exos as a prospective therapeutic strategy for diabetic wound healing stems from their ability to exert precise regulation over the healing process. The primary objective is not merely to accelerate wound closure but to achieve high-quality tissue regeneration. As mentioned previously, Hsu et al. (69) demonstrated that ADSC-Exos promote the proliferation and differentiation of fibroblasts into myofibroblasts by modulating the TGF-β/Smad3 signaling pathway. Persistent TGF-β1 overactivation drives fibrosis by promoting fibroblast-to-myofibroblast differentiation and excessive ECM deposition. This fibrotic response relies heavily on downstream Smad signaling: Smad2 and Smad3 drive pro-fibrotic gene expression, whereas Smad7 acts as a protective negative feedback regulator (72, 73), **Figure 3** shows this mechanism. To halt fibrosis without disrupting essential TGF-β functions, ADSC-Exos offer a precise intervention. Rather than indiscriminately inhibiting the entire pathway, these exosomes deliver specific microRNAs that selectively degrade TGF-β1 mRNA. Song et al. (48) found that ADSC-Exos deliver enriched miR-204-5p to fibroblasts. By directly inhibiting TGF-β1 expression and subsequent Smad2/3 phosphorylation, this microRNA reduces myofibroblast differentiation. The treated models exhibited significantly lower Type I collagen deposition and reduced scar tissue area. A similar mechanism was reported by Liang et al. (28), where ADSC-Exos utilized miR-128-1-5p to target TGF-β1. Blocking this downstream Smad2/3 signaling promotes fibroblast proliferation and migration in a diabetic environment. At the same time, it prevents their differentiation into myofibroblasts. This dual action effectively accelerates wound healing without triggering excessive scar formation. From a clinical standpoint, managing fibrosis in diabetic foot ulcers is fundamentally about balancing scar quality with wound tensile strength. Because the diabetic foot constantly endures weight-bearing loads and shear forces, overly suppressing ECM deposition to prevent scarring can render the nascent tissue structurally fragile. This fragility directly increases the risk of wound dehiscence and recurrent ulceration (2). ADSC-Exos address this specific clinical dilemma. Their mechanism goes beyond simple collagen inhibition; they actively fine-tune the remodeling phase. As a result, the healed tissue achieves the necessary biomechanical strength to handle physical stress, yet avoids the rigidity and functional loss associated with hypertrophic scars. This capacity to deliver both structural durability and healthy tissue architecture makes ADSC-Exos a highly translational alternative to conventional single-target therapies.

## 3 Clinical translation strategies for ADSC-Exos: functional optimization and intelligent delivery

**Figure 4:**
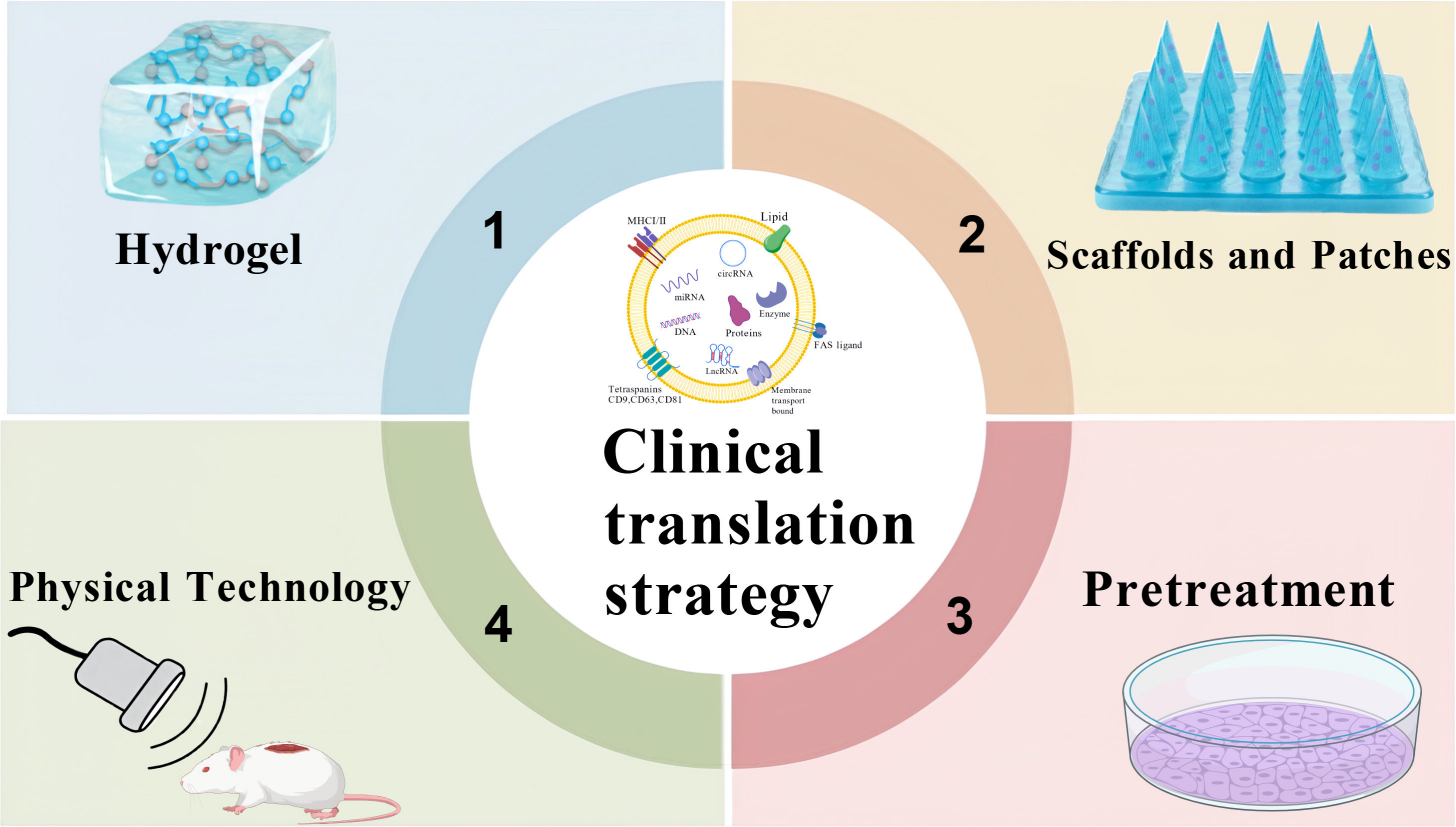
Clinical translation of adipose-derived stem cells exosomes. The clinical application of ADSC-Exos can be advanced through four main approaches: (1) Combination with hydrogel for sustained and localized release; (2) Incorporation into biological scaffolds and patches to provide structural support and targeted delivery; (3) Pretreatment of Cells to Enhance Their Exosomal Function; and (4) Utilization of physically assisted technologies to improve exosome delivery efficiency. (Schematic created with BioGDP).

Although ADSC-Exos demonstrate multiple therapeutic potentials in the treatment of DFU, their clinical translation still faces formidable challenges. The DFU microenvironment is characteristically severe, characterized by local hypoxia, severe oxidative stress, and abnormally high protease activity. Naked exosomes administered directly into the wound bed are highly susceptible to rapid proteolytic degradation and oxidative damage, leading to rapid clearance and difficulty in maintaining effective concentrations (74). To overcome these bottlenecks, current research strategies primarily focus on two major directions: functional optimization and innovation in delivery systems. The former enhances the intrinsic activity of exosomes through methods such as pretreatment, while the latter ensures their targeting and sustained release with the aid of biomaterial technology. This chapter will systematically elaborate on these key technological strategies that promote the transition of ADSC-Exos from basic research to clinical application (**Figure 4**).

### 3.1 Pretreatment strategies

Preconditioning of ADSCs involves placing them in a controlled stress environment to induce the secretion of “enhanced” exosomes that are highly enriched with functional molecules and possess greater targeting specificity. Hypoxic preconditioning and pharmaceutical preconditioning are two currently representative strategies. Hu et al. (75) discovered that hypoxic preconditioned ADSC-derived exosomes significantly promoted the proliferation, migration, and angiogenesis of endothelial cells by delivering circ-Snhg11 and activating the miR-144-3p/NFE2L2/HIF1α pathway. Similarly, Wang et al. (71) experimentally demonstrated that HypADSC-Exos accelerated diabetic foot ulcer healing by carrying specific miRNAs to activate the PI3K/AKT signaling pathway, and that HypADSC-Exos were more effective than exosomes derived from normoxic conditions in promoting high-quality healing of diabetic wounds. Exposure of ADSCs to controlled sub-physiological oxygen tension induces cellular reprogramming, enriching the secreted exosomal cargo with hypoxia-inducible pro-angiogenic effectors. Crucially, this molecular priming confers enhanced functional stability to the vesicles, ensuring the preservation of paracrine signaling within the severely oxygen-deprived wound bed (46, 71, 75). To address the severe angiogenic dysfunction in diabetic wounds, hypoxic preconditioning acts as a direct intervention to promote angiogenesis and accelerate diabetic wound healing. Beyond physical stress, pharmaceutical preconditioning has also been proven to be an effective functional optimization strategy. Wang et al. (76) experimentally confirmed that empagliflozin preconditioned ADSC-derived exosomes, after being taken up by endothelial cells, systematically enhanced the angiogenic capacity of endothelial cells through the PTEN/AKT/VEGF pathway, thereby synergistically promoting the repair and regeneration of diabetic wounds both structurally and functionally.

### 3.2 Advanced delivery systems for precise controlled release

#### 3.2.1 Hydrogel

Hydrogel, as a novel biomaterial, is essentially an insoluble hydrophilic polyurethane polymer. It is widely used in treating DFU wounds due to its moisturizing properties, biocompatibility, and structural similarity to living tissues, which enable hydrogels to create an optimal wound healing environment. Hydrogels are three-dimensional (3D) network structures with high water content, whose hydrophilicity depends on the degree of cross-linking of their polar functional groups. When applied directly to the wound, the 3D network structure facilitates the absorption and retention of moisture. Maintaining a moist wound environment over time helps support gas exchange, cell migration, and tissue regeneration within the wound, thereby promoting the healing process (77–80). Meanwhile, hydrogels do not adhere to the wound bed, allowing for easy application and removal without causing secondary trauma, which makes them an ideal dressing for DFU (81–83). Conventional direct injection therapy often leads to the rapid *in situ* clearance of exosomes, resulting in a short therapeutic window. Utilizing hydrogels addresses this limitation by enabling sustained release, which significantly extends the effective duration of action of the exosomes (74).

Basically, Huldani et al. (84) experimentally confirmed that hyaluronic acid (HA) can provide a three-dimensional structure supporting cell migration and infiltration, and its combined use with ADSC-Exos promoted DFU healing. Zhang et al. (85) were the first to co-load ADSC-Exos with metformin (PEG/Ag/CNT-M+E), demonstrating that the PEG/Ag/CNT-M+E hydrogel synergistically improved microvascular function and ultimately accelerated diabetic wound healing by regulating the mitochondrial fission-ROS-F-actin axis. Zhang et al. (86) successfully developed a multifunctional HAP/OCS/PEG/Ag-E hydrogel with multiple dynamic cross-linking. The hydrogel exhibits excellent self-healing, tissue adhesion, high stretchability, antibacterial activity, and biocompatibility, making it an ideal dressing for diabetic wound care. It can specifically respond to the hyperglycemic/acidic microenvironment of diabetic wounds, enabling intelligent controlled release of ADSC-exosomes. This effectively addresses the issues of rapid *in vivo* clearance and low bioactivity associated with ADSC-exosomes. To combat the high protease activity that typically degrades naked biologics in diabetic wounds, researchers have developed stimuli-responsive smart materials that turn this pathological feature into a therapeutic trigger. For instance, Jiang et al. (87) successfully developed an enzyme-responsive ADSC-Exos@MMP-PEG smart hydrogel. To circumvent non-specific proteolytic degradation, this hydrogel leverages the upregulated matrix metalloproteinases (MMPs) characteristic of the DFU microenvironment. By incorporating MMP-cleavable peptide sequences, the crosslinked polymeric matrix facilitates the stimuli-responsive, on-demand release of encapsulated exosomes. Prior to enzymatic cleavage, the dense 3D network provides critical steric shielding against premature proteolysis. Concurrently, the hydrogel attenuates local oxidative stress by protecting the exosomal lipid bilayer from ROS-induced peroxidation. This structural barrier acts synergistically with the intrinsic antioxidant cargo of the exosomes to effectively scavenge reactive oxygen species, thereby remodeling the pathological wound microenvironment and restoring tissue homeostasis. Wang et al. (88) developed an injectable, self-healing, and antibacterial polypeptide-based FHE hydrogel (F127/OHA-EPL) for chronic wound healing. This hydrogel enables a stimuli-responsive release of adipose-derived mesenchymal stem cell exosomes (ADSC-exos), and their research demonstrates that the sustained release of exosomes, in synergy with the FHE hydrogel, can significantly enhance diabetic wound healing and promote complete skin regeneration.

#### 3.2.2 Biological scaffolds and patches

Acellular biological scaffolds and patches not only inherit the excellent biocompatibility and drug-loading capacity of hydrogels but, more critically, their intrinsic three-dimensional porous structure can provide robust physical support for cell infiltration, tissue regeneration, and vascular ingrowth, thereby achieving structural repair and functional reconstruction of the wound (89, 90). Khalatbary et al. (91) were the first to combine exosomes derived from ADSCs with a three-dimensional microporous amniotic membrane scaffold, demonstrating a synergistic enhancement effect. The amniotic membrane scaffold provided a three-dimensional microenvironment promoting cell migration and angiogenesis; the exosomes modulated inflammation and promoted collagen synthesis and cell proliferation; the combination of both factors was significantly superior to either treatment alone. Furthermore, Talebpour et al. (92) combined Chitosan (CTS) with ADSC-Exos and found that the CTS could provide a microporous structure promoting cell migration and angiogenesis, and its degradation controlled the sustained release of exosomes, prolonging the therapeutic window and significantly improving the treatment outcome. Porcine pericardium is a tissue rich in ECM, readily available, and widely accessible. Liang et al. (93) experimentally demonstrated that in a diabetic mouse wound model, a bilayer acellular porcine pericardial patch loaded with ADSC-Exos significantly promoted wound healing, reduced wound area, and enhanced granulation tissue formation, re-epithelialization, vascularization, and collagen deposition.

#### 3.2.3 Physically assisted technology

In addition to developing advanced delivery materials, directly enhancing the internalization efficiency of exosomes by cells through physical means is also a highly promising strategy for efficacy improvement. Low-intensity pulsed ultrasound (LIPUS) is a type of specific physical energy that is delivered at a low intensity (<3 W/cm2) and outputs in the mode of pulsed waves (94). Research indicates that LIPUS, a non-invasive localized mechanical stimulus, may exert its effects by participating in the regulation of critical cellular processes such as proliferation, differentiation, and apoptosis via intracellular signaling pathways (95, 96). Zhong et al. (97) first demonstrated that LIPUS significantly enhances angiogenesis and tissue repair during diabetic wound healing by promoting the uptake of ADSC-Exos and improving their utilization efficiency, providing a promising new strategy for the treatment of diabetic wounds.

## 4 Challenges and critical perspectives in clinical translation

Although ADSC-Exos demonstrate significant therapeutic efficacy in DFU models, their clinical translation is constrained by systemic bottlenecks. Bridging this gap necessitates a rigorous appraisal of existing methodological limitations, scalable biomanufacturing protocols, *in vivo* biosafety profiles, and evolving regulatory frameworks.

### 4.1 Isolation technologies, characterization, and reproducibility challenges

The absence of standardized isolation and characterization protocols remains a fundamental bottleneck in ADSC-Exos research, driving pervasive irreproducibility and data discrepancies. Conventional separation methodologies—including ultracentrifugation, size exclusion chromatography, and polymer precipitation—impose an inherent trade-off between yield and purity, frequently resulting in vesicular aggregation or the co-isolation of non-exosomal contaminants. Compounding these technical limitations, intrinsic donor heterogeneity and disparate culture microenvironments introduce profound batch-to-batch variability. Mitigating these systemic inconsistencies necessitates rigorous compliance with the Minimal Information for Studies of Extracellular Vesicles guidelines. Standardizing these workflows through quantitative particle profiling, ultrastructural evaluation, and precise validation of transmembrane markers is paramount to guaranteeing the molecular identity, purity, and functional consistency of ADSC-Exos preparations (98, 99).

### 4.2 Large-scale production, quality control, and storage stability

Translating ADSC-Exos into a widely available cell-free therapy necessitates robust, Good Manufacturing Practice -compliant large-scale production. 2D static cultures are insufficient for clinical demands; thus, scalable platforms like 3D bioreactors using hollow fibers or microcarriers must be optimized (100, 101). In tandem with scale-up, establishing stringent quality control metrics and dose standardization remains unresolved. Currently, dosing is arbitrarily based on total protein concentration or particle count, neither of which accurately reflects the therapeutic potency of the encapsulated bioactive cargo (102). Additionally, storage stability poses a critical logistical hurdle. Lyophilization and cryopreservation are common, but repeated freeze-thaw cycles can rupture vesicle membranes and degrade RNA payloads (103). Developing optimal cryoprotectants (such as trehalose) and determining the exact shelf-life of ADSC-Exos under varying temperature conditions are essential to maintain their biological efficacy during distribution (104).

### 4.3 Safety profiles: immunogenicity, off-target effects, and tumorigenic risks

Although ADSC-Exos circumvent many risks associated with live stem cell therapies, their safety profile is not entirely benign. Firstly, regarding immunogenicity, while exosomes exhibit low immunogenic profiles, repeated administrations of allogeneic ADSC-Exos may still elicit immune clearance or hypersensitivity reactions over time. Secondly, off-target effects remain a concern, Exosomes can be internalized through multiple non-selective pathways including phagocytosis, macropinocytosis, clathrin-mediated endocytosis and caveolin-dependent endocytosis without relying on specific receptors, thus entering various cell types indiscriminately. Moreover, Exosomes are highly heterogeneous consisting of exosomes and microvesicles with distinct sizes and compositions, and different Exosomes subsets show different biodistribution profiles. Finally, passive biodistribution determined by Exosomes size and administration route further reduces specific accumulation in target tissues and cells, all of which collectively contribute to off-target effects (105). Most critically, the tumorigenic risk must be carefully evaluated. Exosomes serve as key mediators of intercellular communication in the tumor microenvironment. They mediate signaling by delivering functional miRNAs, proteins, lipids and metabolites, directly regulating tumor cell proliferation, apoptosis and cell cycle progression. They also activate pro−angiogenic and pro−metastatic signaling pathways including VEGF, ERK/MAPK and Wnt, promote endothelial cell proliferation, angiogenesis and extracellular matrix remodeling, and enhance tumor cell invasion, migration and distant colonization. The potent pro-angiogenic and pro-proliferative properties of ADSC-Exos—while highly beneficial for wound healing—could theoretically accelerate the growth of dormant or undiagnosed malignancies in patients (106, 107). Long-term safety tracking in large animal models is essential to fully elucidate these risks before human trials (108).

### 4.4 Navigating the regulatory approval pathways

The regulatory landscape for stem cell-derived EVs remains ambiguous and highly complex. Regulatory bodies such as the US Food and Drug Administration and the European Medicines Agency typically classify exosomes as biological products or Advanced Therapy Medicinal Products, subjecting them to rigorous Chemistry, Manufacturing, and Controls requirements (102, 109). The inherent heterogeneity of exosomes complicates the definition of the “active pharmaceutical ingredient” and pharmacokinetics profiling. Establishing a clear, standardized regulatory pathway specifically tailored for EV-based therapies is a prerequisite. This entails defining acceptable tolerance limits for batch variability, standardizing potency assays, and reaching a consensus on preclinical toxicity evaluation frameworks (110). Collaboration among researchers, clinicians, and regulatory agencies is urgently needed to establish these guidelines and accelerate the clinical translation of ADSC-Exos.

## 5 Conclusion and future prospects

ADSC-Exos present a viable cell-free therapeutic strategy for DFUs. They facilitate tissue repair by alleviating oxidative stress, resolving local inflammation, stimulating angiogenesis, and regulating ECM remodeling to prevent fibrosis. Furthermore, integrating these vesicles with functional delivery systems, improves their *in vivo* stability and enables sustained, targeted release within the diabetic wound environment.

To translate this therapy from preclinical models into practical clinical use, future research must focus on addressing four critical gaps in current knowledge and methodology:

1. Engineered Exosomes: Subsequent studies should explore the genetic or chemical modification of parent ADSCs, as this approach holds great promise for optimizing exosome function. By engineering exosomes to carry concentrated therapeutic cargoes and display homing peptides, we can significantly enhance their accumulation at the site of ischemic DFUs and thereby improve their therapeutic efficacy.
2. Biomarker-Guided Therapy: Ischemic DFUs exhibit considerable clinical heterogeneity, which underscores the need to identify reliable predictive biomarkers—such as specific microRNA profiles in wound exudates. These biomarkers will allow for effective stratification of patient subpopulations and enable real-time monitoring of wound healing progress, helping to tailor treatment strategies to individual patients.
3. Rigorous Clinical Trial Design: A critical bottleneck in advancing this therapy is the severe paucity of human clinical data. Moving forward, priority should be given to well-designed randomized controlled trials, which are essential for establishing optimal dosing regimens and objectively measuring the clinical benefits of this therapy compared to standard-of-care treatments, such as negative pressure wound therapy.
4. Preclinical Standardization: To ensure batch-to-batch consistency of exosomes, it is necessary to establish Good Manufacturing Practice-compliant protocols for their isolation and storage. Additionally, therapeutic efficacy must be thoroughly validated in metabolically relevant large animal models—such as diabetic porcine models—to better replicate the clinical conditions of human DFUs and support subsequent clinical translation.

Tackling these translational hurdles is the next logical step. Once standardized and validated in robust human trials, ADSC-Exos have the true potential to move beyond the laboratory and offer a definitive, cell-free treatment for diabetic foot ulcers.

## Glossary

ADSC-Exos: Adipose-Derived Stem Cell Exosomes
Col-I: Collagen Type I
CTS: Chitosan
DFU: Diabetic Foot Ulcer
ECM: Extracellular Matrix
EPC: Endothelial Progenitor Cell
EVs: Extracellular Vesicles
FGF4: Fibroblast Growth Factor 4
GelMA: Gelatin Methacryloyl
HA: Hyaluronic Acid
HG: High Glucose
HIF-1α: Hypoxia-Inducible Factor-1α
HUVECs: Human Umbilical Vein Endothelial Cells
HypADSC-Exos: Hypoxia-preconditioned Adipose-Derived Stem Cell Exosomes
LIPUS: Low-Intensity Pulsed Ultrasound
MDA: Malondialdehyde
MMPs: Matrix Metalloproteinases
MSCs: Mesenchymal Stem Cells
Nrf1: Nuclear Respiratory Factor 1
ROS: Reactive Oxygen Species
SIRT3: Sirtuin 3
Smad: Small mother against decapentaplegic
SOD: Superoxide Dismutase
SOD2: Superoxide Dismutase 2
STAT3: Signal Transducer and Activator of Transcription 3
T-AOC: Total Antioxidant Capacity
α-SMA: Alpha-Smooth Muscle Actin
